# Supplementary material for: A Looping-Based Model for Quenching Repression
Source: PLoS Comput Biol. 2017 Jan 13;13(1):e1005337. doi: 10.1371/journal.pcbi.1005337 (PMC5279812; doi:10.1371/journal.pcbi.1005337)
Supplement: S1 File — (PDF) [file pcbi.1005337.s001.pdf]

1 Extended Materials and Methods

1.1 Neglecting torsional degree of freedom

The contribution of the torsional degree of freedom to the elastic energy of the ith link is:

$$\beta E_i^{twist} = c\left(\Omega_i - \Omega_0\right)^2, \tag{1}$$

where $\beta = \left(k_b T\right)^{-1}$, Ω_0 is the relaxed twisting angle of the DNA chain (the native twist of ≈1.81 radian per nm), Ω_i is the twist angle of link i relative to the previous link $i-1$, and c is the torsional rigidity constant.

The DNA double-helix retains memory of the minimal energy relative phase between two links provided the distance between them is on the order of the torsional persistence length (∼260 bp [10]). There are two relative phases that are relevant to our problem. First, the relative phase between the two chain termini, which can be taken into account in looping conditions. Second, the relative phase between the chain origin and the link at which the protrusion is located, which determines the orientation of the protrusion relative to the looping volume $\delta\mathbf{r}$ (see Figure 1). The phases of the chain termini, that are separated in this work by many torsional persistence lengths, are completely uncorrelated, and thus the relative phase of the two termini is uniformly distributed ([1] Figure 1B). Any looping condition on the relative phase would merely decrease the number of looped configurations based on the uniformly distributed phase of the final terminus without altering the effect of the protrusion (i.e. the protrusion would eliminate the same portion of looped configurations, only there would be fewer of these configurations to begin with). This is why we did not include a torsional restriction in the looping conditions, and why we do not retain phase information for the distal chain terminus. Regarding the relative phase between the chain origin and the protrusion link, the distance between these two links is much smaller than the torsional persistence length, therefore, there is minimal deviation from the native twist of the DNA, and the relative orientation of the protrusion with respect to the looping volume $\delta\mathbf{r}$ is dictated by the intervening length K. We chose to neglect the torsional information for these links so that we could decouple the dependence of the eclipsing effect on K from its dependence on the orientation of the protrusion (also dependent on K). We checked both extreme protrusion orientations (in-phase and out-of-phase with $\delta\mathbf{r}$, see Figure 2A) and found that while the model adequately explains the behavior of the effect for both orientations, the effect is maximal for the in-phase orientation. Thus our model is valid for K sufficiently small for the dsDNA to retain its torsional rigidity (K smaller than the torsional persistence length ∼260 bp [10]). This is the range of parameters discussed in the paper (see Figure 2B).

1.2 Numerical implementation overview

In order to evaluate the looping probabilities, we used a Monte-Carlo algorithm to generate a large number ($10^8 - 10^{10}$) of self-avoiding DNA chains. The Monte-Carlo simulation was written in CUDA C++, and was executed on two NVIDIA GeForce GTX TITAN cards.

Derivation of the sampling probability and Rosenbluth weights

The probability density of a chain configuration $\mathbf{x}$ is given by:

$$\pi\left(\mathbf{x}\right) = \frac{\exp\left(-\beta E\left(\mathbf{x}\right)\right)}{Z} = \frac{\exp\left(-\beta E\left(\mathbf{x}\right)\right)}{\int \exp\left(-\beta E\left(\mathbf{x}\right)\right) \mathrm{d}\mathbf{x}}, \tag{2}$$

where $\mathbf{x}$ is a single chain configuration, corresponding to $\{\theta, \phi\}_N$ in equation (1) in the manuscript, $E\left(\mathbf{x}\right) \equiv E\left(\{\theta,\phi\}_N\right)$ in the same equation, and

$$Z = \int \exp\left(-\beta E\left(\mathbf{x}\right)\right) \mathrm{d}\mathbf{x}, \tag{3}$$

is the partition function. Following the notations in [6], for a chain $\mathbf{x}$ we defined a sequence of auxiliary distributions $\pi_t\left(\mathbf{x}_t\right)$, $t = 1, 2, ..., N-1$ for the sub-chain (partial sample) $\mathbf{x}_t = \left(x_1, x_2, ..., x_t\right)$ with t links:

$$\pi_t\left(\mathbf{x}_t\right) = \frac{\exp\left(-\beta E\left(\mathbf{x}_t\right)\right)}{\int \exp\left(-\beta E\left(\mathbf{x}_t\right)\right) \mathrm{d}\mathbf{x}_t} = \frac{\exp\left(-\beta E\left(\mathbf{x}_t\right)\right)}{Z_t}, \tag{4}$$

where Z_t is the partition function of a self-avoiding worm-like chain with t links. The marginal distribution of the partial sample $\mathbf{x}_{t-1} = \left(x_1, ..., x_{t-1}\right)$ is

$$\pi_t\left(\mathbf{x}_{t-1}\right)=\int\pi_t\left(\mathbf{x}_{t-1},x_t\right)dx_t=\frac{\int\exp\left(-\beta E\left(\mathbf{x}_{t-1},x_t\right)\right)\mathrm{d}x_t}{Z_t}=\frac{\exp\left(-\beta E\left(\mathbf{x}_{t-1}\right)\right)}{Z_t}\int\exp\left(-\beta E_t\left(x_t|\mathbf{x}_{t-1}\right)\right)\mathrm{d}x_t, \quad (5)$$

where $E_t\left(x_t|\mathbf{x}_{t-1}\right)$ is the potential energy of the tth link x_t given $t-1$ previous links $\mathbf{x}_{t-1}$. The second transition is possible due to the fact that the energy of the chain can be broken to contributions from individual links:

$$\exp\left(-\beta E\left(\mathbf{x}_t\right)\right)=\prod_i\exp\left(-\beta E\left(x_i|\mathbf{x}_{i-1}\right)\right). \quad (6)$$

The conditional distribution is therefore given by:

$$\pi_t\left(x_t|\mathbf{x}_{t-1}\right)=\frac{\pi_t\left(x_t,\mathbf{x}_{t-1}\right)}{\pi_t\left(\mathbf{x}_{t-1}\right)}=\frac{\frac{\exp(-\beta E(\mathbf{x}_t))}{Z_t}}{\frac{\exp(-\beta E(\mathbf{x}_{t-1}))}{Z_t}\int\exp\left(-\beta E_t\left(x_t|\mathbf{x}_{t-1}\right)\right)\mathrm{d}x_t}=\frac{\exp\left(-\beta E_t\left(x_t|\mathbf{x}_{t-1}\right)\right)}{\int\exp\left(-\beta E_t\left(x_t|\mathbf{x}_{t-1}\right)\right)\mathrm{d}x_t}. \quad (7)$$

We set the incremental sampling distribution $g_t\left(x_t|\mathbf{x}_{t-1}\right)$ in the growth method for sampling the tth link x_t given $\mathbf{x}_{t-1}$ exactly equal to the conditional distribution $\pi_t\left(x_t|\mathbf{x}_{t-1}\right)$:

$$g_t\left(x_t|\mathbf{x}_{t-1}\right)=\frac{\exp\left(-\beta E_t\left(x_t|\mathbf{x}_{t-1}\right)\right)}{\int\exp\left(-\beta E_t\left(x_t|\mathbf{x}_{t-1}\right)\right)\mathrm{d}x_t}. \quad (8)$$

The importance weight of link t is now given by:

$$\begin{aligned}\tilde{w}_t\left(\mathbf{x}\right) &= \frac{\pi_t\left(\mathbf{x}_t\right)}{\pi_{t-1}\left(\mathbf{x}_{t-1}\right)g_t\left(x_t|\mathbf{x}_{t-1}\right)}=\frac{\frac{\exp(-\beta E(\mathbf{x}_t))}{Z_t}}{\frac{\exp(-\beta E(\mathbf{x}_{t-1}))}{Z_{t-1}}\frac{\exp(-\beta E_t(x_t|\mathbf{x}_{t-1}))}{\int\exp(-\beta E_t(x_t|\mathbf{x}_{t-1}))\mathrm{d}x_t}} \\ &= \frac{Z_{t-1}}{Z_t}\exp\left(-\beta E_t\left(x_t|\mathbf{x}_{t-1}\right)\right)\frac{\int\exp\left(-\beta E_t\left(x_t|\mathbf{x}_{t-1}\right)\right)\mathrm{d}x_t}{\exp\left(-\beta E_t\left(x_t|\mathbf{x}_{t-1}\right)\right)}=\frac{Z_{t-1}}{Z_t}\int\exp\left(-\beta E_t\left(x_t|\mathbf{x}_{t-1}\right)\right)\mathrm{d}x_t \quad (9)\end{aligned}$$

And the total importance weight is given by:

$$\tilde{w}\left(\mathbf{x}\right)=\prod_{t=1}^N\tilde{w}_t\left(\mathbf{x}\right)=\frac{1}{Z}\prod_{t=1}^N\int\exp\left(-\beta E_t\left(x_t|\mathbf{x}_{t-1}\right)\right)\mathrm{d}x_t \quad (10)$$

Due to the fact that we do not know Z (or the individual Z_t) we define the weights up to a multiplicative constant and use a biased estimator for the chain properties (see Section 3.1):

$$w_t\left(\mathbf{x}\right) = \int\exp\left(-\beta E_t\left(x_t|\mathbf{x}_{t-1}\right)\right)\mathrm{d}x_t \quad (11)$$

and

$$w\left(\mathbf{x}\right)=\prod_{t=1}^N w_t\left(\mathbf{x}\right). \quad (12)$$

The probability to draw a chain $\mathbf{x}$ sequentially is:

$$g\left(\mathbf{x}\right)=\prod_{t=1}^N g_t\left(x_t|\mathbf{x}_{t-1}\right)=\prod_{t=1}^N\frac{\exp\left(-\beta E_t\left(x_t|\mathbf{x}_{t-1}\right)\right)}{\int\exp\left(-\beta E_t\left(x_t|\mathbf{x}_{t-1}\right)\right)\mathrm{d}x_t}=\frac{\exp\left(-\beta E\left(\mathbf{x}\right)\right)}{w\left(\mathbf{x}\right)}. \quad (13)$$

The expectation value of a property v is computed from an ensemble of m chains as:

$$\langle\hat{v}\rangle=\frac{\sum_{i=1}^m w\left(\mathbf{x}^{(i)}\right)v\left(\mathbf{x}^{(i)}\right)}{\sum_{i=1}^m w\left(\mathbf{x}^{(i)}\right)}, \quad (14)$$

where $\mathbf{x}^{(i)}$ is the ith chain in the ensemble. This estimate is asymptotically unbiased, but is biased for finite m.

Sampling procedure

Each self-avoiding worm-like chain (SAWLC) in our ensemble was grown one link at a time by selecting the link's orientation according to the incremental sampling distribution $g_t(x_t|\mathbf{x}_{t-1})$ (8):

$$g_t(x_t|\mathbf{x}_{t-1}) = \frac{\exp[a(1-\cos\theta_i)]\,\Theta_i^{hw}(\{\theta_i,\phi_i\})}{\int \exp[a(1-\cos\theta_i)]\,\Theta_i^{hw}(\{\theta_i,\phi_i\})\,\mathrm{d}\theta_i \mathrm{d}\phi_i}, \quad (15)$$

where

$$\Theta_i^{hw}(\{\theta_i,\phi_i\}) = \begin{cases} 0 & \text{joint } i \text{ overlaps with one or more joints } 0..(i-1) \\ 1 & \text{otherwise} \end{cases}. \quad (16)$$

We sampled the link orientation angles $(\cos\theta_i, \phi_i)$ using inversion sampling [2]. Inversion sampling of a single random variable X from a probability distribution function $(PDF(X))$ is carried out by, first, integrating the $PDF(X)$ over the entire range of X, resulting in a cumulative distribution function $(CDF(X))$. Then, a random number y in the range $(0,1)$ is generated from a continuous uniform distribution. Finally, the desired variable value x is extracted from the inverse function of the $CDF(X)$ at y: $x = CDF^{-1}(y)$. We first consider the case of the chain without the excluded volume constraint. Namely, we compute the inverse of the following function:

$$CDF(\cos\theta_i) = \frac{\int_{-1}^{\cos\theta_i} \mathrm{d}\cos\theta \int_{-\pi}^{\pi} \mathrm{d}\phi\, e^{-a(1-\cos\theta)}}{\int_{-1}^{1} \mathrm{d}\cos\theta \int_{-\pi}^{\pi} \mathrm{d}\phi\, e^{-a(1-\cos\theta)}}, \quad (17)$$

which allows us to draw $\cos\theta_i$. Then, we generate ϕ_i from a uniform distribution over the entire range $\phi_i \epsilon [0, 2\pi)$. When we reintroduce the hard-wall potential, the above integral now changes to:

$$CDF(\cos\theta_i) = \frac{\int_{-1}^{\cos\theta_i} \mathrm{d}\cos\theta \int_{allowed\,\phi\,for\,\theta} \mathrm{d}\phi\, e^{-a(1-\cos\theta)}}{\int_{-1}^{1} \mathrm{d}\cos\theta \int_{allowed\,\phi\,for\,\theta} \mathrm{d}\phi\, e^{-a(1-\cos\theta)}}. \quad (18)$$

As a result, the integral over ϕ is no longer over the entire $[0, 2\pi)$ range, as some directions in space cannot be assumed by the new link due to the excluded volume constraint. Thus, for each possible θ angle of the new link, there is a different range of ϕ values that are allowed. Since there can be more than one colliding object, the set of allowed values for ϕ_i need not be one continuous range. Rather, these values can be organized into $m \geq 1$ consecutive ranges $[r_{i,0}^a, r_{i,0}^b], ..., [r_{i,m-1}^a, r_{i,m-1}^b]$, such that

$$\phi_i^{tot} = \sum_{j=0}^{m-1} \left(r_{i,j}^b - r_{i,j}^a\right) \leq 2\pi. \quad (19)$$

Thus, for each θ_i the ϕ_i angle can be sampled from a step-wise uniform distribution:

$$P_i^{\phi}(\theta_i,\, \phi_i \epsilon (0, 2\pi)) = \frac{\sum_{j=0}^{m-1} \Theta\left(\phi_i - r_{i,j}^a\right) \Theta\left(r_{i,j}^b - \phi_i\right)}{\phi_i^{tot}}. \quad (20)$$

We computed the $CDF(\cos\theta_i)$ numerically, using the adaptive Gaussian integration method [9], and subsequently inverted it.

1.3 Simulation details

Simulation parameters Simulated ensembles varied in size depending on the desired accuracy, with the smallest ensembles containing 10^8 samples and the largest containing 5×10^9. The length of the generated chains in most cases (except for the cases described in the section titled "Looping at chain ends vs. looping in the middle of the chain" in the manuscript) was limited to 10^4 bp, which is 850-970 links (100-250 base-pair sized links for the enhancer region and 750-720 w =4.6 nm sized links for the region between the enhancer and the promoter). As can

be seen in Figure 1 this two stage procedure has no effect on looping probability for chains longer than ∼500 bp, and no effect on looping probability ratio.

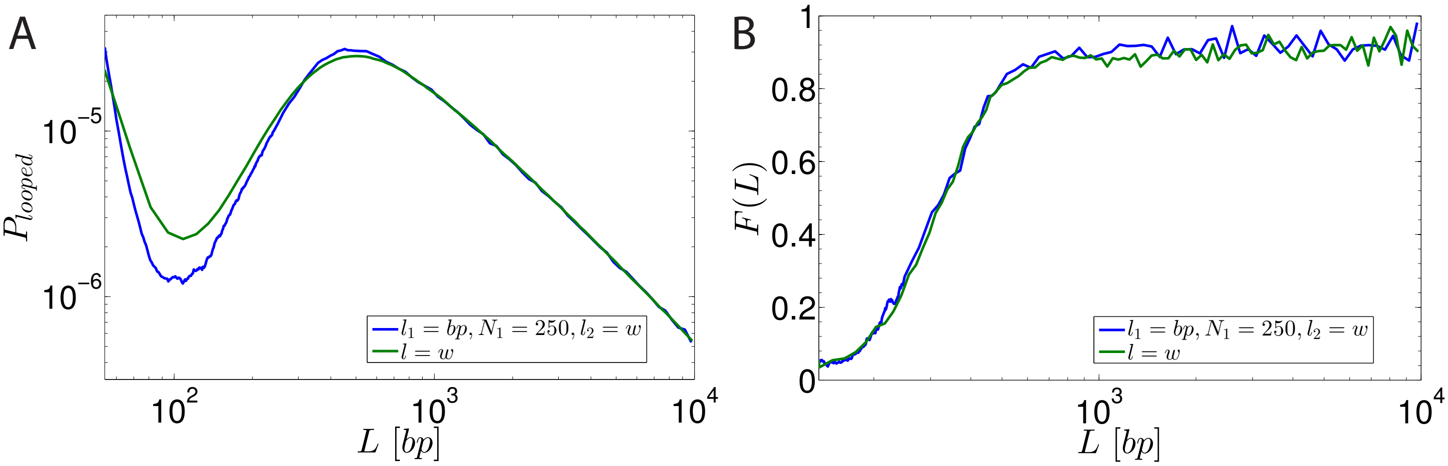

Figure 1: Comparison between chains simulated in two stages (250 short links of base-pair length, followed by links of length w=4.6 nm) and chains simulated with long ($l = w$) links only. A) Comparison between the looping probabilities of bare polymer chains. B) Comparison between loopingprobability ratios of polymer chains with protrusions.

The running time changed significantly, depending on the location of the protrusion and the number of links generated, with an average sampling rate of approximately 10^7 samples per minute.Figure 2B required 197 separate simulation runs, each containing 2×10^8 complete chains of length 10^4 bp, with protrusions at various locations $K = (15..240)$ bp in increments of 15 bp, and various protrusion radii $R_o = (1.7..23.8)$ nm in increments of 1.7 nm. An additional simulation was run without a protrusion, containing 5×10^9 chains in order to estimate the probability of looping without a protrusion. The large amount of samples in this simulation was needed to improve the accuracy of $F(L)$ estimation, as the probability of looping with a protrusion was divided by the probability of looping without a protrusion.

For the plots in Figure 3 we run two simulations in which the protrusion was positioned in a static location at $(41.45\,\text{nm})\,\hat{u}_0$, and its radius was $R_0 = 23\,\text{nm}$. Each simulation contained 2×10^9 samples. In one simulation we removed chain-chain interactions, leaving only chain-static object interactions, while in the second simulation we introduced both chain-chain and chain-static object interactions.

For the plots in Figure 5 we ran two simulations in which we added a leading segment of one Kuhn length b. Each simulation contained 5×10^8 samples. In one simulation we positioned the protrusion on the leading segment (upstream) at a distance of 95 bp from the looping region. In the other simulation we positioned the protrusion after the looping region (downstream) at a distance of 95 bp from it. We ran an additional simulation of 5×10^9 samples without a protrusion in order to compute $F(L)$.

***D. melanogaster eve* 3/7 enhancer** For the plot in Figure 8 we ran a simulation modeling the 3/7 enhancer in *D. melanogaster*. The structure of the enhancer was modeled based on [11]. In particular, the locations of the protein complexes were 16, 43, 68, 88, 112, 268, 310, 342, 409, 446 and 483 bp. The locations of the two dStat activators were 202 and 323 bp, and the location of the Zld activator was 416 bp. The chain was simulated as 512 base-pair sized links, followed by 237 links of size $w = 4.6$ nm, followed by 150 base-pair sized links, giving a total of 3900 bp between both ends of the chain. We used $R_{dStat} = 3.04$ nm, $R_{Zld} = 3.75$ nm and three protein complex sizes of $R_o = 2.38$ nm, $R_o = 3.59$ nm and $R_o = 5.83$ nm. The activator and protein complex protrusions were located around the chain axis according to the native DNA twist of $2\pi/10.5$.

We ran 4 simulations in total: one simulation with only the three activator protrusions present, containing 5×10^9 samples, and three simulations with all activators present as well as different sized protrusions corresponding to the three values of R_o, containing 2×10^8 samples each.

Looping in the middle of the chain simulation In order to compute the probability of looping for a generic chain, where the loop now takes place between two widely separated internal links of the chain, we extended the

original length of the chain L by two segments of 10 Kuhn lengths ($10b$) from each end of the chain. We denote the links added before the looping region (chain origin) as links $-10b...0$ and the links added after the chain terminus as $N+1,...,N+10b$. The procedure of the chain generation was changed in the following way. First, we generated a leading trail of $10b$ links prior to the location of the enhancer, computing its cumulative Rosenbluth weight as usual. We proceeded to generate the rest of the links of the chain as before. When a new link in the range $n \in [10b, N+10b]$ was generated, its cumulative Rosenbluth weight $W_n \equiv \prod_{i=-10b}^{n} w_i$ was assigned to link $n-10b$. This effectively resulted in an enhancer-promoter region of length $n-10b$, with two flanking regions of length $10b$ each, as required. This scheme allowed us to generate the looping probability data for all lengths of chains in a single run as before.

Confining sphere simulation In the case of chains confined by a sphere, the simulation process was broken into several stages:

1. The initial links of the chain were generated as before, with the bounding sphere of the links generated thus far being continuously computed.
2. When the size of the bounding sphere reached the confining size, the sphere's location was frozen.
3. The frozen bounding sphere acted as an additional hard-wall potential for all links generated henceforth.

The computation of a minimal sphere encompassing spheres is a very demanding task [3]. Computation of a minimal bounding sphere encompassing points is slightly less demanding, but nevertheless very costly. In our case, however, we do not require precision in the determination of the bounding sphere, especially since the results show that the size of the confining sphere has no effect on the looping probability ratio. Therefore, we used a very simple algorithm for the bounding sphere computation:

1. We begin with a sphere with the center at the origin $\mathbf{c} = (0, 0, 0)$ and radius $R = 0$.
2. When a new joint $\mathbf{x}_i$ is added to the chain, we check whether it is inside the sphere.
   (a) If $|\mathbf{x}_i - \mathbf{c}| \leq R$, nothing happens.
   (b) If $|\mathbf{x}_i - \mathbf{c}| > R$ the sphere is enlarged and moved in the direction of the new link, to accommodate it:

$$d = |\mathbf{x}_i - \mathbf{c}| - R \tag{21}$$

$$R = R + \frac{d}{2} \tag{22}$$

$$\mathbf{c} = \mathbf{c} + \frac{d}{2}\left(\frac{\mathbf{x}_i - \mathbf{c}}{|\mathbf{x}_i - \mathbf{c}|}\right) \tag{23}$$

3. Repeat step 2 until R is bigger or equal to the radius of the confining sphere.

The result of this algorithm is a confining sphere of the target size around an initial segment of the chain. For a freely jointed chain this method produces a bounding sphere with radius 5%-25% larger than the radius of the minimal bounding sphere, with an average of 20%, independent of the number of links generated (data not shown). Thus, the placement of the confining sphere is not ideal, with the chain link that caused the sphere freeze touching the surface of the sphere, and the origin of the sphere distanced $0.1R \leq s \leq 0.5R$ away from the sphere's surface. We partially compensate for this problem by advancing the sphere center $\mathbf{c}$ in the direction $\frac{\mathbf{x}_i - \mathbf{c}}{|\mathbf{x}_i - \mathbf{c}|}$ by $\min\{0.15R,\ s\}$. While this is a slight improvement, the placement of the frozen sphere is still far from ideal. However, since our results show no dependency at all on the size of the bounding sphere, this inaccuracy is inconsequential.

1.4 Computation of F_∞ and its error estimation

To compute the value of F_∞ for a specific simulation we averaged the values of $F(L)$ for $L \geq 10b$. According to the model and the numerical simulations themselves, at this separation and onward $F(L)$ was constant. We denote the first link satisfying this condition as N_{10b} and the last link of the chain as N. We considered each pair $F(N_i)$, $F(N_j)$ independent for all $i \neq j$. Thus:

$$F_\infty = \frac{1}{(N - N_{10b} + 1)} \sum_{n=N_{10b}}^{N} F(l \cdot n) \tag{24}$$

where l is the length of the link. In our simulations $N - N_{10b} + 1 = 480$. The standard error of the mean is

$$\delta F_\infty = \sqrt{\frac{\sum_{n=N_{10b}}^{N} (F(l \cdot n) - F_\infty)^2}{N - N_{10b} + 1}}. \tag{25}$$

In our plots we plot the error bars with $1.96\delta F_\infty$.

2 Blob sizes for model organisms

Chromosomal DNA is compacted into a globular state. For low enough volume fractions it can be described as a semi-dilute polymer solution. This semi-dilute solution may be pictured as a system of blobs of characteristic size ξ. Inside each blob, the chain behaves as an isolated macromolecule with excluded volume, and different blobs are statistically independent of one another. The blob size can be roughly estimated as [4]:

$$\xi \sim b\Phi^{-3/4}, \tag{26}$$

where Φ is the polymer volume fraction, which for DNA is organism-dependent. The number of consecutive chain Kuhn lengths located in a blob is roughly given by [4]:

$$g \sim \Phi^{-5/4}. \tag{27}$$

Therefore for a volume fraction of $\Phi \approx 0.01$ in *E. coli* [5] and $b = 100$ nm for bare DNA [12] we get a blob size of $\xi_{E.coli} \sim 3200$ nm and $bg_{E.coli} \sim 320b \approx 90$ kbp inside a blob. Similarly, for a volume fraction of $\Phi \approx 0.015$ in *D. melanogaster* [5] and $b = 200$ nm for chromatin [12] we get an even larger blob size of $\xi_{D.melanogaster} \sim 4600$ nm. These rough estimates suggest that for realistic volume fractions, a typical blob will contain many Kuhn lengths of polymer.

3 Estimation of the bias in the expectation value of the looping probability

3.1 Bias origin

Given the distribution of $\mathbf{x}$, $\pi(\mathbf{x})$, the mean value of a function $v(\mathbf{x})$ is evaluated as:

$$E_\pi [v(\mathbf{x})] = \int v(\mathbf{x}) \pi(\mathbf{x}) \, \mathrm{d}\mathbf{x}. \tag{28}$$

There is an inherent bias in estimating the mean value with importance sampling. The bias results from the fact that $E_\pi [v(\mathbf{x})]$ is estimated from m samples by a biased estimator

$$\langle \hat{v} \rangle = \frac{\sum_{i=1}^{m} w\left(\mathbf{x}^{(i)}\right) v\left(\mathbf{x}^{(i)}\right)}{\sum_{i=1}^{m} w\left(\mathbf{x}^{(i)}\right)}, \tag{29}$$

instead of the unbiased one (40):

$$\langle \widetilde{v} \rangle = \frac{1}{Z} \frac{1}{m} \sum_{i=1}^{m} w\left(\mathbf{x}^{(i)}\right) v\left(\mathbf{x}^{(i)}\right), \tag{30}$$

where $w\left(\mathbf{x}^{(i)}\right)$ is the importance weight of the configuration i (12) and Z is the partition function of $\pi'(\mathbf{x}) \equiv Z\pi(\mathbf{x})$ (3). The unbiased estimator's expectation value is equal to the mean value of the corresponding function (40):

$$E_g [\langle \widetilde{v} \rangle] = E_\pi [v(\mathbf{x})], \tag{31}$$

where $g(\mathbf{x})$ is the sampling distribution (13). However, the unbiased estimator cannot be used, since it requires the knowledge of $\pi(\mathbf{x})$, while usually we only know the distribution $\pi(\mathbf{x})$ up to a constant Z.

3.2 Bias analysis

In this section we follow the derivations of [7] and adapt them to the SAWLC case. The true expectation value of a property v is computed from $\pi(\mathbf{x})$:

$$\langle v\rangle = \int v(\mathbf{x})\,\pi(\mathbf{x})\,\mathrm{d}\mathbf{x} = \frac{\int v(\mathbf{x})\exp(-\beta E(\mathbf{x}))\,\mathrm{d}\mathbf{x}}{Z}. \tag{32}$$

The partition function Z (3) and the mean value $\langle v\rangle$ are estimated from the numerical ensemble of m samples by:

$$\hat{Z} = \frac{1}{m}\sum_{i=1}^{m} w\left(\mathbf{x}^{(i)}\right) \tag{33}$$

and (29):

$$\langle\hat{v}\rangle = \frac{\sum\limits_{i=1}^{m} w\left(\mathbf{x}^{(i)}\right) v\left(\mathbf{x}^{(i)}\right)}{\sum\limits_{i=1}^{m} w\left(\mathbf{x}^{(i)}\right)}. \tag{34}$$

We shall continue to use $\langle\hat{v}\rangle$ for the mean value estimated from m samples, to distinguish it from $\langle v\rangle$ the true mean value of the ensemble.
First, we show that the estimator of $\hat{Z}$ (33) is unbiased. For an estimated property $\hat{u}$ we can compute the expectation value (integrating over all possible configurations, taking into account the probability to draw those configuration numerically $g(\mathbf{x})$) as:

$$E[\hat{u}] \equiv E_g[\hat{u}] = \int u(\mathbf{x})\,g(\mathbf{x})\,\mathrm{d}\mathbf{x}. \tag{35}$$

Thus we can compute the expectation value of $\hat{Z}$. Plugging (13) into (33), since $\mathbf{x}^{(i)}$ are independent $E\left[w\left(\mathbf{x}^{(i)}\right)\right] = E\left[w\left(\mathbf{x}^{(j)}\right)\right]$:

$$E\left[\hat{Z}\right] = \frac{1}{m}\sum_{i=1}^{m} E\left[w\left(\mathbf{x}^{(i)}\right)\right] = E[w(\mathbf{x})] = \int w(\mathbf{x})\frac{\exp(-\beta E(\mathbf{x}))}{w(\mathbf{x})}\mathrm{d}\mathbf{x} = \int \exp(-\beta E(\mathbf{x}))\,\mathrm{d}\mathbf{x} = Z. \tag{36}$$

The variance of $\hat{Z}$ is related to the variance of w, because $\hat{Z}$ is just the average of m independent values of w:

$$var\left(\hat{Z}\right) = \frac{1}{m}var(w) = \frac{1}{m}\left(E\left[w^2\right] - E[w]^2\right) = \frac{1}{m}\left(\int w(\mathbf{x})^2\frac{\exp(-\beta E(\mathbf{x}))}{w(\mathbf{x})}\mathrm{d}\mathbf{x} - Z^2\right) = \frac{\int w(\mathbf{x})\exp(-\beta E(\mathbf{x}))\,\mathrm{d}\mathbf{x} - Z^2}{m}. \tag{37}$$

For a parameter v we define an unbiased (up to a constant Z) estimator of $\langle v\rangle$:

$$R = \frac{1}{m}\sum_{i=1}^{m} v\left(\mathbf{x}^{(i)}\right) w\left(\mathbf{x}^{(i)}\right) \tag{38}$$

so that

$$\langle\hat{v}\rangle = \frac{\sum\limits_{i=1}^{m} w\left(\mathbf{x}^{(i)}\right) v\left(\mathbf{x}^{(i)}\right)}{\sum\limits_{i=1}^{m} w\left(\mathbf{x}^{(i)}\right)} = \frac{R}{\hat{Z}}. \tag{39}$$

Then the expectation value of R:

$$E[R] = \int v(\mathbf{x})\,w(\mathbf{x})\frac{\exp(-\beta E(\mathbf{x}))}{w(\mathbf{x})}\mathrm{d}\mathbf{x} = \int v(\mathbf{x})\exp(-\beta E(\mathbf{x}))\,\mathrm{d}\mathbf{x} = Z\langle v\rangle. \tag{40}$$

Thus the estimator $\langle\hat{v}\rangle$ is asymptotically unbiased. For finite m we expand $\langle\hat{v}\rangle$ around $\left(R_0, \hat{Z}_0\right) = \left(E[R], E\left[\hat{Z}\right]\right)$:

$$\langle \hat{v} \rangle = \frac{E[R]}{E\left[\hat{Z}\right]} + \frac{1}{E\left[\hat{Z}\right]}\left(R - E[R]\right) - \frac{E[R]}{E\left[\hat{Z}\right]^2}\left(\hat{Z} - E\left[\hat{Z}\right]\right) - \frac{1}{E\left[\hat{Z}\right]^2}\left(R - E[R]\right)\left(\hat{Z} - E\left[\hat{Z}\right]\right) + \frac{E[R]}{E\left[\hat{Z}\right]^3}\left(\hat{Z} - E\left[\hat{Z}\right]\right)^2 + \text{higher order terms.} \tag{41}$$

We take the expectation value of $\langle \hat{v} \rangle$. The second and third terms vanish, we plug in (36),(40) and we're left with:

$$\begin{aligned} E[\langle \hat{v} \rangle] &= \langle v \rangle + \frac{1}{Z^2}\left(\langle v \rangle E\left[\left(\hat{Z} - Z\right)^2\right] - E\left[\left(R - E[R]\right)\left(\hat{Z} - E\left[\hat{Z}\right]\right)\right]\right) \\ &= \langle v \rangle + \frac{1}{Z^2}\left(\langle v \rangle E\left[\hat{Z}^2\right] - \langle v \rangle Z^2 - E\left[R\hat{Z}\right] + E[R]\,Z\right) \\ &= \langle v \rangle + \frac{1}{Z^2}\left(\langle v \rangle E\left[\hat{Z}^2\right] - \langle v \rangle Z^2 - E\left[R\hat{Z}\right] + Z\langle v \rangle Z\right) \\ &= \langle v \rangle + \frac{1}{Z^2}\left(\langle v \rangle E\left[\hat{Z}^2\right] - E\left[R\hat{Z}\right]\right). \end{aligned} \tag{42}$$

But

$$E\left[\hat{Z}^2\right] = var\left(\hat{Z}\right) + E\left[\hat{Z}\right]^2 = \frac{\int w(\mathbf{x}) \exp(-\beta E(\mathbf{x}))\, \mathrm{d}\mathbf{x} - Z^2}{m} + Z^2 = \frac{1}{m}\int w(\mathbf{x}) \exp(-\beta E(\mathbf{x}))\, \mathrm{d}\mathbf{x} + \frac{m-1}{m} Z^2, \tag{43}$$

where we used (37),(36). In addition:

$$\begin{aligned} E\left[R\hat{Z}\right] &= \frac{1}{m^2} E\left[\sum_{k=1}^{m} v\left(\mathbf{x}^{(k)}\right) w\left(\mathbf{x}^{(k)}\right) \sum_{i=1}^{m} w\left(\mathbf{x}^{(i)}\right)\right] \\ &= \frac{1}{m^2} E\left[\sum_{k=1}^{m} v\left(\mathbf{x}^{(k)}\right) w\left(\mathbf{x}^{(k)}\right)^2\right] + \frac{1}{m^2} E\left[\sum_{k \neq i}^{m} \sum_{i=1}^{m} v\left(\mathbf{x}^{(k)}\right) w\left(\mathbf{x}^{(k)}\right) w\left(\mathbf{x}^{(i)}\right)\right] \end{aligned} \tag{44}$$

$$\frac{1}{m^2} E\left[\sum_{k=1}^{m} v\left(\mathbf{x}^{(k)}\right) w\left(\mathbf{x}^{(k)}\right)^2\right] = \frac{1}{m}\int v(\mathbf{x})\, w(\mathbf{x})^2 \frac{\exp(-\beta E(\mathbf{x}))}{w(\mathbf{x})} \mathrm{d}\mathbf{x} = \frac{1}{m}\int v(\mathbf{x})\, w(\mathbf{x}) \exp(-\beta E(\mathbf{x}))\, \mathrm{d}\mathbf{x} \tag{45}$$

$$\frac{1}{m^2} E\left[\sum_{k=i}^{m} \sum_{i=1}^{m} v\left(\mathbf{x}^{(k)}\right) w\left(\mathbf{x}^{(k)}\right) w\left(\mathbf{x}^{(i)}\right)\right] = \frac{m(m-1)}{m^2} E\left[v\left(\mathbf{x}^{(k)}\right) w\left(\mathbf{x}^{(k)}\right)\right] E\left[w\left(\mathbf{x}^{(i)}\right)\right] = \frac{m-1}{m} E[R]\, E[w] = \frac{m-1}{m} Z^2 \langle v \rangle \tag{46}$$

$$E\left[R\hat{Z}\right] = \frac{1}{m}\int v(\mathbf{x})\, w(\mathbf{x}) \exp(-\beta E(\mathbf{x}))\, \mathrm{d}\mathbf{x} + \frac{m-1}{m} Z^2 \langle v \rangle. \tag{47}$$

Thus, plugging (43) and (47) into (42):

$$E[\langle \hat{v} \rangle] = \langle v \rangle + \frac{1}{mZ^2}\left(\langle v \rangle \int w(\mathbf{x}) \exp(-\beta E(\mathbf{x}))\, \mathrm{d}\mathbf{x} - \int v(\mathbf{x})\, w(\mathbf{x}) \exp(-\beta E(\mathbf{x}))\, \mathrm{d}\mathbf{x}\right) \tag{48}$$

$$= \langle v \rangle - \frac{1}{mZ^2}\left(\int \left(v(\mathbf{x}) - \langle v \rangle\right) w(\mathbf{x}) \exp(-\beta E(\mathbf{x}))\, \mathrm{d}\mathbf{x}\right). \tag{49}$$

Hence the bias:

$$\boxed{B_m = E[\langle \hat{v} \rangle] - \langle v \rangle = -\frac{1}{mZ^2}\left(\int \left(v(\mathbf{x}) - \langle v \rangle\right) w(\mathbf{x}) \exp(-\beta E(\mathbf{x}))\, \mathrm{d}\mathbf{x}\right)}. \tag{50}$$

Similar to [7] we obtained that the bias scales as m^{-1}, thus we expect it to be very small for the typical $m = 10^8$ we used in our simulations.

3.3 Practical estimation of the bias

While (50) is a very nice formula, it is not practical, as it requires integration over the entire phase space of a chain. [8] develops a practical estimate of the bias, which we adapt to the SAWLC case following his derivations. For p independent runs of m chains each, the covariance of $\langle \hat{v} \rangle$ and $\hat{Z}$ can be computed as:

$$cov\left(\langle\hat{v}\rangle,\hat{Z}\right)=\frac{1}{p}\sum_{j=1}^{p}\langle\hat{v}\rangle_j\hat{Z}_j-\frac{1}{p^2}\left(\sum_{j=1}^{p}\langle\hat{v}\rangle_j\right)\left(\sum_{j=1}^{p}\hat{Z}_j\right). \tag{51}$$

However, by definition

$$cov\left(\langle\hat{v}\rangle,\hat{Z}\right) = E\left[\langle\hat{v}\rangle\hat{Z}\right]-E\left[\langle\hat{v}\rangle\right]E\left[\hat{Z}\right]. \tag{52}$$

Using (39) and 40:

$$E\left[\langle\hat{v}\rangle\hat{Z}\right]=E\left[\frac{R}{\hat{Z}}\hat{Z}\right]=E\left[R\right]=\langle v\rangle Z \tag{53}$$

while from (36):

$$E\left[\langle\hat{v}\rangle\right]E\left[\hat{Z}\right]=E\left[\langle\hat{v}\rangle\right]Z. \tag{54}$$

Therefore

$$cov\left(\langle\hat{v}\rangle,\hat{Z}\right)=\left(\langle v\rangle-E\left[\langle\hat{v}\rangle\right]\right)Z. \tag{55}$$

Thus:

$$\boxed{B_m=E\left[\langle\hat{v}\rangle\right]-\langle v\rangle=-\frac{cov\left(\langle\hat{v}\rangle,\hat{Z}\right)}{Z}} \tag{56}$$

where the $cov\left(\langle\hat{v}\rangle,\hat{Z}\right)$ is estimated from p independent runs, and Z is estimated by $\hat{Z}$ and further refined as

$$Z\approx\frac{1}{p}\sum_{j=1}^{p}\hat{Z}_j. \tag{57}$$

3.4 Estimation of the looping probability bias

We applied the procedure described in the previous section to the estimation of looping probability bias. The smallest ensemble that we used in this work contained 10^8 chains. We performed $p=50$ runs with $m=10^8$ chains in each run, for maximal length $L=10^4$ bp. We used 51 and 56 to estimate the bias. As can be seen in Figure 2 the bias in the looping probability estimation is smaller than the standard deviation of the looping probability estimation by at least 4 orders of magnitude.

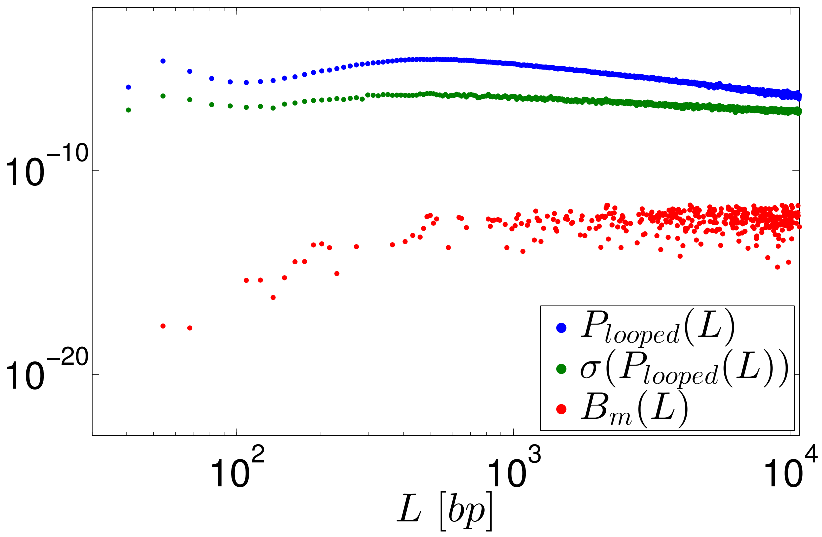

Figure 2: Comparison between the bias in estimation of the looping probability (red) and the standard deviation of the looping probability (green) as a function of generated chain length. The looping probability for a sample run is shown in blue. The estimation is done using $p = 50$ runs with $m = 10^8$ chains in each run, for maximal chain length of $L = 10^4$ bp.

We repeated the bias estimation and standard deviation estimations for the simulations including a confining sphere. Both the bias and the standard deviation were significantly smaller than the looping probability (exhibiting similar behavior to Figure 2) for $R \geq 250$ nm. For $R = 125$ nm our simulations are no longer valid for $L \geq 4400$ bp, due to an increase in standard deviation to the level of the looping probability. The estimated bias reaches the levels of the looping probability and the standard deviation only at higher values of L.

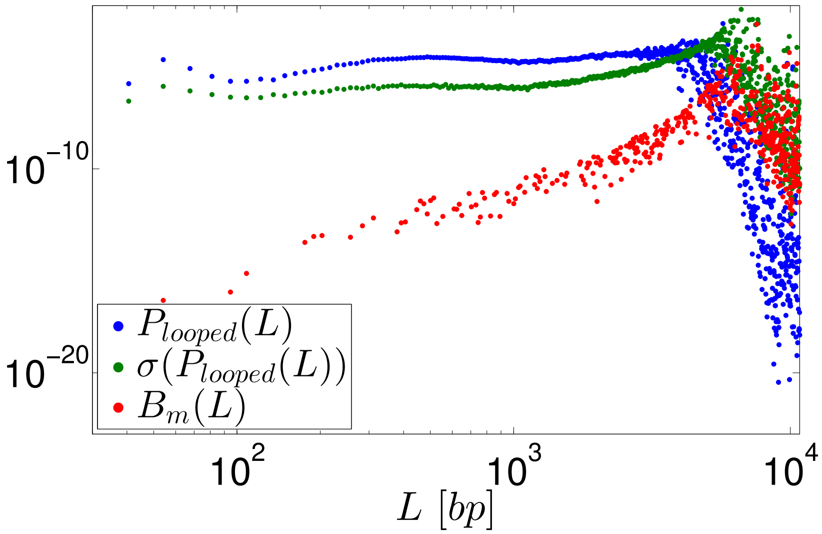

Figure 3: Comparison between the bias in estimation of the looping probability (red) and the standard deviation of the looping probability (green) as a function of generated chain length for chains confined by a sphere with radius $R = 125$ nm. The looping probability for a sample run is shown in blue. The estimation is done using $p = 40$ runs with $m = 2 \times 10^7$ chains in each run, for maximal chain length of $L = 10^4$ bp.

References

[1] Roee Amit, Hernan G. Garcia, Rob Phillips, and Scott E. Fraser. Building enhancers from the ground up: A synthetic biology approach. *Cell*, 146(1):105–118, July 2011.

[2] Luc Devroye. *Non-uniform random variate generation.* Springer-Verlag, New York, 1986.

[3] Kaspar Fischer. *Smallest enclosing balls of balls. Combinatorial structure & algorithms.* PhD thesis, ETH, 2005.

[4] Pierre-Gilles Gennes. *Scaling Concepts in Polymer Physics.* Cornell University Press, Ithaca, NY, November 1979.

[5] Jonathan D. Halverson, Jan Smrek, Kurt Kremer, and Alexander Y. Grosberg. From a melt of rings to chromosome territories: the role of topological constraints in genome folding. *Reports on Progress in Physics*, 77(2):022601, 2014.

[6] Jun S. Liu. *Monte Carlo Strategies in Scientific Computing.* Springer, New York, February 2009.

[7] Frank L. McCrackin. Weighting methods for Monte-Carlo calculation of polymer configurations. *Journal of Research of the National Bureau of Standards, Section B: Mathematical Sciences*, 76B(3-4):193, July 1972.

[8] Frank L. McCrackin, Jacob Mazur, and Charles M. Guttman. Monte Carlo Studies of Self-Interacting Polymer Chains with Excluded Volume. I. Squared Radii of Gyration and Mean-Square End-to-End Distances and Their Moments. *Macromolecules*, 6(6):859–871, November 1973.

[9] Wilhelm M. Pieper. Recursive gauss integration. *Communications in Numerical Methods in Engineering*, 15(2):77–90, 1999.

[10] T. R. Strick, D. Bensimon, and V. Croquette. Micro-mechanical measurement of the torsional modulus of DNA. *Genetica*, 106(1-2):57–62, 1999.

[11] Paolo Struffi, Maria Corado, Leah Kaplan, Danyang Yu, Christine Rushlow, and Stephen Small. Combinatorial activation and concentration-dependent repression of the Drosophila even skipped stripe 3+7 enhancer. *Development*, 138(19):4291–9, Oct 2011.

[12] Marian Walhout, Marc Vidal, and Job Dekker, editors. *Handbook of Systems Biology: Concepts and Insights.* Academic Press, London, 1 edition edition, December 2012.
